# Genetic association analysis of coronary heart disease by profiling gene-environment interaction based on latent components in longitudinal endophenotypes

**DOI:** 10.1186/1753-6561-3-s7-s86

**Published:** 2009-12-15

**Authors:** C Charles Gu, Wei (Will) Yang, Aldi T Kraja, Lisa de las Fuentes, Victor G Dávila-Román

**Affiliations:** 1 Division of Biostatistics, Washington University School of Medicine, 660 South Euclid Avenue, Box 8067, St. Louis, Missouri 63110, USA; 2 Department of Genetics, Washington University School of Medicine, 660 South Euclid Avenue, Box 8067, St. Louis, Missouri 63110-1093, USA; 3 Division of Statistical Genomics, Department of Genetics, 4444 Forest Park Boulevard, Box 8506, St. Louis, MO 63108, USA; 4 Cardiovascular Imaging and Clinical Research Core Laboratory, Cardiovascular Division, Department of Medicine, Washington University School of Medicine, 660 South Euclid Avenue, Campus Box 8086, St. Louis, Missouri 63110, USA

## Abstract

Studies of complex diseases collect panels of disease-related traits, also known as secondary phenotypes or endophenotypes. They reflect intermediate responses to environment exposures, and as such, are likely to contain hidden information of gene-environment (G × E) interactions. The information can be extracted and used in genetic association studies via latent-components analysis. We present such a method that extracts G × E information in longitudinal data of endophenotypes, and apply the method to repeated measures of multiple phenotypes related to coronary heart disease in Genetic Analysis Workshop 16 Problem 2. The new method identified many genes, including *SCNN1B *(sodium channel nonvoltage-gated 1 beta) and *PKP2 *(plakophilin 2), with potential time-dependent G × E interactions; and several others including a novel cardiac-specific kinase gene (*TNNI3K*), with potential G × E interactions independent of time and marginal effects.

## Background

"Endophenotypes" refer to the host of measurements representing physiologic indicators, biochemical assays, and responses to challenges, or the latent components extracted from such data [1]. When derived properly, the latent traits lay more proximal to the causal genotypes than do clinical phenotypes, and thus, provide potentially meaningful but otherwise unobserved context of gene-environment (G × E) interaction. Several recent studies report positive findings with endophenotypes in genetic analysis of complex diseases [2,3]. Our group recently developed a supervised statistical learning approach for multivariate analysis (SLAM) that uses latent component methods to extract meaningful latent traits for association studies. We have applied this method to the study of hypertension and hypertensive heart disease [4]. The method worked well to identify meaningful latent traits of hypertensive heart disease that led to detection of significant genotype × phenotype associations that were missed by analyses of measured clinical phenotypes.

The repeated measures of multiple coronary heart disease (CHD)-related phenotypes from Genetic Analysis Workshop (GAW) 16 Problem 2 are ideal for testing this approach to identify genetic variants that interact with the environment in the development of CHD. For example, systolic and diastolic blood pressure represent continuous and independent risk factors for CHD events [5], and increased blood pressure was associated with pathologic remodeling of the left ventricle [6]. Studies have identified dyslipidemia as a major cause of CHD; therapies that lower serum low-density lipoprotein cholesterol reduce CHD risk [7]. Indeed, metabolic syndrome, which represents a constellation of major risk factors including abdominal obesity, atherogenic dyslipidemia, elevated blood pressure, insulin resistance, and prothrombotic and proinflammatory states, has been shown to increase risk of CHD [8]. Repeated measures on these CHD endophenotypes and their environmental risks (e.g., cigarette smoking) contain valuable information about underlying mechanisms of G × E interactions that is biologically relevant to the development and/or modulation of CHD.

In the present study, we explore such underlying mechanisms using latent components (referred to as "G × E context") extracted by an extension of the SLAM approach to analyze the multivariate longitudinal data in GAW 16 Problem 2.

## Methods

### Data adjustment and quality control

Samples in the "Offspring Cohort" (of the Framingham Heart Study) and data from Visits 1, 3, 5, and 7 were used in this study. The primary phenotype of interest was CHD event, and the data on ten variables including CHD endophenotypes (body mass index, three lipids, blood pressures, and glucose) and environmental covariates (age at visit, cigarette smoking, and alcohol use) were used for latent component analysis. The endophenotypes were checked for normality and outliers, and log-transformed when necessary; this was followed by centering the variables by sex to remove unwanted confounding. The residuals were used as input for all downstream analyses. We used the genome-wide dense single-nucleotide polymorphisms (SNPs) dataset provided for Problem 2 (~550,000 SNPs typed by Affymetrix GeneChip^® ^Human Mapping 500 k Array Set). Quality of each SNP array was checked first for low call rate (<95%) and/or abnormal heterozygosity (<0.25 or >0.3); then each SNP on the array was checked for its minor allele frequency (MAF < 0.05), missing rate in the sample (>5%), and deviation from Hardy-Weinberg equilibrium (*p *< 10^-6^). Problematic individuals and SNPs were moved from further analysis.

### Identifying latent G × E context

Latent component analysis (also known as factor analysis) aims to effectively reduce the number of dimensions (variables) for analysis while minimizing the loss of information. Conventional factor analysis, of which the well known principal-component analysis is a special case, seeks to reduce the dimensionality by expressing the original variables as linear combinations of a smaller number of independent, Gaussian, latent variables (components). However, it tends to neglect meaningful structural information such as clustering in data, which often requires non-Gaussian components and proper treatment of higher order of moments than covariance and correlations. The method of independent-component analysis (ICA) overcomes this problem by treating observed traits as a mixture of underlying components that are more likely independent, non-Gaussian, and with less complexity than observed ones. It identifies such latent components by maximizing a measure of multivariate non-Gaussianity of linear combinations of original variables [9]. The SLAM approach is built on ICA using supervised validation of extracted components followed by consensus analysis of validated components to identify robust and biologically-meaningful latent traits [4]. For the present study, we extend SLAM by applying multi-level ICA to facilitate analysis of longitudinal multivariate data.

#### Time-dependent longitudinal G × E context

We first applied a four-component ICA at each visit on the ten selected variables to identify latent components that define potentially meaningful underlying G × E context for CHD. Then, correlations between the derived independent components (ICs) at consecutive visits were examined and those with strongest correlations were concatenated to form four *longitudinal *latent components (LLCs). We hypothesized that each (most) of the derived LLCs represents a particular G × E mechanism (context) for the development of the disease, and can be used as a *derived "environment" variable *for teasing out potential G × E interactions. This was verified by logistic regression of CHD on each extracted LLC at every time point (adjusted for age at visit) to evaluate LLCs as a predictor of CHD, followed by regression of each LLC on genome-wide SNP genotypes to assess its genetic content.

#### Summary time-independent G × E context

A second-level two-component ICA was then performed on each LLC over the four time points (i.e., visits). For each LLC, we anticipated that one such derived component will capture the main signal of the time-course, while the other absorbs remaining signal and noise. Identification of the signal component was assisted by a clinical expert knowledgeable in the component's capacity in predicting CHD risks. In the end, this procedure derives time-independent components (TICs) to capture the time course of G × E interactions in the context defined by each LLC. Note that familial relationship is ignored during ICA extraction and is accounted for in the downstream association analysis.

### Profiling G × E interactions

The identified latent components provide different G × E context useful for teasing out potential G × E interactions. The LLCs have repeated measures at each visit, and can be used to facilitate a focused search for SNPs with potential time-dependent G × E interactions. Logistic regression of CHD status was carried out first against SNP genotypes and LLC values, then by an expanded model including the term for SNP × LLC interaction. These analyses were done for each visit, and results were aggregated to spot trends of time-dependent interactions. Finally, the TICs represent summary profiling of time-course of G × E interactions for CHD. The detection of SNPs with potential time-independent G × E interactions for CHD was then achieved by testing for significant SNP × TIC interactions using logistic regression. In all regression analyses, the generalized estimating equation approach was used to adjust for correlations among family members in the sample.

## Results

Samples of 2584 individuals in the “Offspring Cohort” were used in this study. After removing samples missing at least 1 visit (n=403) and those without GWAS data (n=187), we performed genotype quality control and excluded 33 samples due to low call rate (n=22), abnormal heterozygosity (n=5) and population outliers (n=6). A total of 1961 individuals were used in the analyses reported below. To achieve normality, log-transformations were applied to triglyceride, blood glucose, cigarettes smoked per day, and alcohol use per week.

### Time-dependent G × E interactions

Repeated measures from Visits 1, 3, 5, and 7 were used to extract time-dependent G × E context, the LLCs for CHD. The extended SLAM procedure was used to extract four LLCs as described previously.

We first tested each LLC as a predictor of CHD. Table 1 shows the results by logistic regression of CHD on LLC at each visit. With the exception of LLC1, the derived components LLC2-LLC4 were all significant predictors of CHD. Both LLC2 and LLC3 are highly significant predictors of CHD at the early two visits, while LLC2 was also fairly significant at Visit 7. LLC4 seemed to have captured a complementary axis that became significant predictor of CHD at later visits when the average age of the Offspring Cohort reaches 53 to 60 years old. Note that LLC1 absorbs remaining variation in the data, and may still contain some G × E information (as confirmed below). We then examined genetic association of SNPs with each LLC by longitudinal regression. There were a number of SNPs with *p *≤ 0.05, but few achieved high significance. Most notable ones were all associated with LLC3 (21 SNPs in 9 genes with *p *≤ 1 × 10^-4^, data not shown). These results supported the idea that the LLCs may be used as a derived "environment" variable for teasing out potential G × E interactions.

**Table 1:** Logistic regression of CHD on LLCs

LLC	Visit 1	Visit 3	Visit 5	Visit 7
1	0.093742	0.128905	0.189618	0.073004
2	**0.000056^a^**	**0.000023**	0.375683	**0.010654**
3	**0.000115**	**0.007251**	0.140016	0.466008
4	0.333980	**0.040160**	**0.000080**	**0.007423**

^a^Bold font indicates *p*-values ≤ 0.05.

We then tested for SNP × LLC interactions in an expanded model including the interaction terms (CHD~age+LLC+SNP+SNP*LLC). A total of 76 SNPs in 59 genes were found having 96 significant interactions with various LLCs at different time points, at a significance level of α = 1 × 10^-6^. A majority of these SNP × LLC interactions (63/96 ≈ 66%) were detected in the middle two visits, and close to half were interacting with LLC1. Many of the genes detected are relevant to CHD. In Table 2, we displayed some representatives in details. Some of them, including *SCNN1B *and *PKP2*, are well known candidate genes of CHD.

**Table 2:** Representative candidate genes and SNPs that were detected with significant SNP × LLC interactions (*p *≤ 10^-6^)

				Visit			
				
SNP ID	Chr	MAF	Gene	1	3	5	7
SNP_A-4199078	2	0.07	*IL1RN*		LLC1		
SNP_A-1788738	4	0.06	*KLF3*				LLC4
SNP_A-1978322	4	0.06	*TACR3*	LLC3			
SNP_A-2260338	5	0.06	*FTMT*	LLC2			
SNP_A-2031704	5	0.15	*NSD1*		LLC4		
SNP_A-1987480	6	0.15	*TRDN*	LLC4			
SNP_A-2090526	6	0.06	*CITED2*		LLC2		
SNP_A-4217972	10	0.07	*KCNMA1*		LLC2		
SNP_A-4272586	12	0.05	*PIK3C2G*				LLC1
SNP_A-1961226	12	0.10	*PKP2*		LLC1	LLC1	
SNP_A-4222134	13	0.15	*IRS2*			LLC2	
SNP_A-2306682	16	0.07	*SCNN1B*			LLC4	
SNP_A-2019383	21	0.07	*PSMG1*		LLC1	LLC1	
SNP_A-2051756	22	0.07	*MB*			LLC3	

### Time-independent G × E Interactions

**Figure 1:**
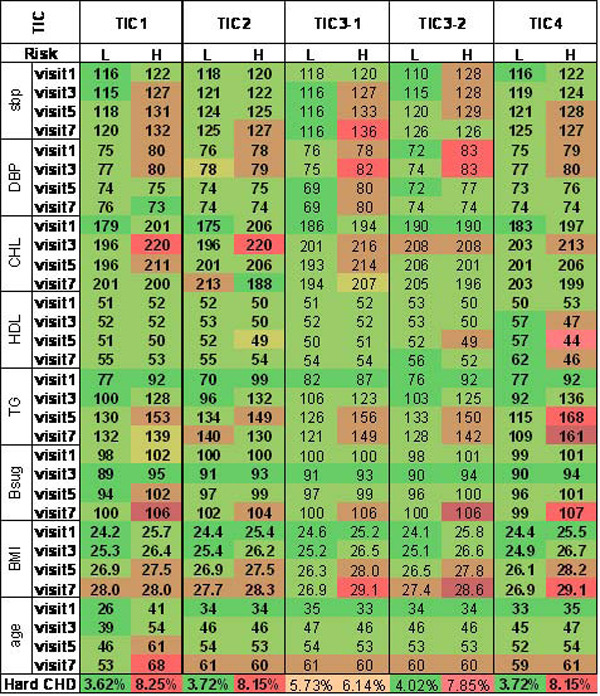
**Validation of selected TICs**. Displayed are mean values of eight clinical indicators of CHD and prevalence of hard CHD in "high-risk" (H) and "low-risk" (L) groups as defined by the TIC in question. sbp, systolic blood pressure; DBP, diastolic blood pressure; CHL, cholesterol; HDL, high-density lipoprotein; TG, triglyceride; Bsug, blood sugar; BMI, body mass index. The component that distinguishes the two groups well are selected as the "signal" component that captures the time-course of LLC relevant to CHD. Bright red colors indicate higher risk for CHD and darker green colors indicate lower risk.

As described previously, we then performed second-level ICA on each LLC to extract the component that captures main signal of the time-course underlying the LLC. These components are denoted TIC1 to TIC4. Note that we include LLC1 in the second-level ICA analysis, assuming that there may still be G × E information hidden in this "noise" component. Results of validation analyses of the derived TICs are shown in Figure 1, where their capacity in predicting CHD risks are evident in eight clinical indicators of CHD including blood pressures, lipids, blood sugar, body mass index, and age. It is interesting to note that both of the two components (denoted as TIC3-1 and TIC3-2 in Figure 1) derived from LLC3 may qualify as a "signal" component, although TIC3-2 did better in predicting CHD events.

To study effects of potential interactions between SNPs and so-derived TICs, we carried out logistic regression of CHD status on SNP genotypes and an interaction term for SNP × TIC, using two types of models: ones that include main effect of TICs and ones without. We hypothesized that SNPs for which the two models produced similar significance levels imply possibility of "pure" SNP × TIC effects. At a significance level of α = 10^-5^, we displayed in Table 3 genes containing such SNPs, for which the inclusion of main effects of TICs did not substantially change the significance level of detected interactions between the SNPs and the time-independent latent components. The maximum of the *p*-values of the two models were shown for the most significant SNPs in each gene. Among these, the cardiac-specific kinase *TNNI3K *interacts specifically with cardiac troponin I and has been found to protect the myocardium from ischemic injury [10].

**Table 3:** Genes containing ≥ 1 SNPs with "pure" SNP × TIC interactions for CHD (*α *= 10^-5^), and *p*-values for most significant SNPs and their MAFs

Chr	MAF	Gene	TIC1	TIC2	TIC3	TIC4	No. significant SNPs
1	0.052	*TNNI3K*				1.59 × 10^-6^	1
3	0.113	*CNBP*				8.73 × 10^-6^	3
4	0.060	*PCDH7*		4.76 × 10^-7^			1
5	0.066	*PARP8*				8.27 × 10^-6^	1
6	0.160	*TRDN*				3.83 × 10^-6^	3
8	0.237	*EIF3H*				1.78 × 10^-6^	2
8	0.158	*SGCZ*	2.25 × 10^-6^				1
9	0.085	*FREQ*				4.58 × 10^-6^	1
10	0.053	*FAS*			1.16 × 10^-7^		1
15	0.061	*GABRG3*				3.00 × 10^-6^	1
15	0.085	*TJP1*				6.01 × 10^-6^	2
16	0.313	*ARHGAP17*	9.18 × 10^-6^				2

## Discussion

In this study, we showed that the SLAM approach can be extended by including a novel method of two-level latent component analysis to address the challenge of analyzing multivariate longitudinal data of the correlated phenotypes, endophenotypes, covariates, and environmental factors typically found in genome-wide association studies of complex diseases such as CHD. Repeated measures from Visits 1, 3, 5, and 7 from GAW16 Problem 2 were used to extract LLCs (longitudinal latent components) that represents differential age- (visit-) dependent risks. The second-level analysis of LLCs extracted time-independent components (TICs) and captured variants with "pure" SNP × TIC interactions. The method seemed to have worked well in teasing out variants with promising G × E interactions in CHD, by analyses in derived context that potentially homogenize samples according underlying G × E mechanisms. Note that medication uses were not directly modeled because their effects should be reflected in the measured endophenotypes. The derivation of the longitudinal latent components (LLC) may benefit from more rigorous mathematical treatment, e.g., survival analysis of time to events of CHD. Finally, further characterization of G × E mechanisms will require identifying the right environment variables after the extended SLAM analysis, followed by explicit modeling of G × E interactions, and will be the topic of our future studies.

## List of abbreviations used

CHD: Coronary heart disease; G × E: Gene-environment interaction; GAW: Genetic Analysis Workshop; IC: Independent components; LLCs: Longitudinal latent components; MAF: Minor allele frequency; SLAM: Supervised statistical learning approach for multivariate analysis; SNP: Single-nucleotide polymorphism; TIC: Time-independent component.

## Competing interests

The authors declare that they have no competing interests.

## Authors' contributions

CCG developed the concept and method, participated in analysis, drafted and revised the manuscript, and gave final approval for publication; WY performed analysis and help drafted the manuscript; ATK participated in analysis and manuscript development; LdlF participated in analysis and manuscript development; VGD-R and LdlF revised the manuscript critically.

## References

[B1] Gottesman II Gould TD The endophenotype concept in psychiatry: etymology and strategic intentions Am J Psych 2003 160 636 645 10.1176/appi.ajp.160.4.636 12668349

[B2] Almasy L Blangero J Endophenotypes as quantitative risk factors for psychiatric disease: rationale and study design Am J Med Genet 2001 105 42 44 10.1002/1096-8628(20010108)105:1<42::AID-AJMG1055>3.0.CO;2-9 11424994

[B3] Pan WH Lynn KS Chen CH Wu YL Lin CY Chang HY Using endophenotypes for pathway clusters to map complex disease genes Genet Epidemiol 2006 30 143 154 10.1002/gepi.20136 16437587

[B4] Gu CC Flores HR de Las Fuentes L Davila-Roman VG Enhanced detection of genetic association of hypertensive heart disease by analysis of latent phenotypes Genet Epidemiol 2008 32 528 538 10.1002/gepi.20269 18435473

[B5] Lewington S Clarke R Qizilbash N Peto R Collins R Age-specific relevance of usual blood pressure to vascular mortality: a meta-analysis of individual data for one million adults in 61 prospective studies Lancet 2002 360 1903 1913 10.1016/S0140-6736(02)11911-8 12493255

[B6] Huang P Kraja AT Tang W Hunt SC North KE Lewis CE Devereux RB de Simone G Arnett DK Rice T Rao DC Factor relationships of metabolic syndrome and echocardiographic phenotypes in the HyperGEN study J Hypertens 2008 26 1360 1366 18551011 10.1097/HJH.0b013e3282ffdc80 PMC2663576

[B7] Expert Panel on Detection, Evaluation, and Treatment of High Blood Cholesterol in Adults Executive Summary of The Third Report of The National Cholesterol Education Program (NCEP) Expert Panel on Detection, Evaluation, And Treatment of High Blood Cholesterol In Adults (Adult Treatment Panel III) JAMA 2001 285 2486 2497 10.1001/jama.285.19.2486 11368702

[B8] Gami AS Witt BJ Howard DE Erwin PJ Gami LA Somers VK Montori VM Metabolic syndrome and risk of incident cardiovascular events and death: a systematic review and meta-analysis of longitudinal studies J Am Coll Cardiol 2007 49 403 414 10.1016/j.jacc.2006.09.032 17258085

[B9] Hyvärinen A Oja E Independent component analysis: algorithms and applications Neural Netw 2000 13 411 430 10.1016/S0893-6080(00)00026-5 10946390

[B10] Lai ZF Chen YZ Feng LP Meng XM Ding JF Wang LY Ye J Li P Cheng XS Kitamoto Y Monzen K Komuro I Sakaguchi N Kim-Mitsuyama S Overexpression of TNNI3K, a cardiac-specific MAP kinase, promotes P19CL6-derived cardiac myogenesis and prevents myocardial infarction-induced injury Am J Physiol Heart Circ Physiol 2008 295 H708 H716 10.1152/ajpheart.00252.2008 18552163

